# Electrical neurostimulation in glaucoma with progressive vision loss

**DOI:** 10.1186/s42234-022-00089-9

**Published:** 2022-03-31

**Authors:** Carl Erb, Sophie Eckert, Pia Gindorf, Martin Köhler, Thomas Köhler, Lukas Neuhann, Thomas Neuhann, Nadja Salzmann, Stefanie Schmickler, Jens Ellrich

**Affiliations:** 1 Augenklinik Wittenbergplatz, Berlin, Germany; 2 Medizentrum Eckert, Neu-Ulm, Germany; 3 Gemeinschaftspraxis Salzmann/Köhler, Hannover, Germany; 4 MVZ Prof. Neuhann GmbH, Munich, Germany; 5 Augen-Zentrum-Nordwest, Ahaus, Germany; 6 Medical Faculty, Friedrich-Alexander-University, D-91052 Erlangen, Germany

**Keywords:** Neuromodulation, Neuroprotection, Optic nerve, Perimetry, Restoration

## Abstract

**Background:**

The retrospective study provides real-world evidence for long-term clinical efficacy of electrical optic nerve stimulation (ONS) in glaucoma with progressive vision loss.

**Methods:**

Seventy glaucoma patients (45 to 86 y) with progressive vision loss despite therapeutic reduction of intraocular pressure (IOP) underwent electrical ONS. Closed eyes were separately stimulated by bipolar rectangular pulses with stimulus intensities up to 1.2 mA sufficient to provoke phosphenes. Ten daily stimulation sessions within 2 weeks lasted about 80 min each. Right before ONS at baseline (PRE), vision loss was documented by static threshold perimetry and compared to the same assessment approximately 1 year afterwards (POST). Mean defect (MD) was defined as primary outcome parameter. Perimetries with a reliability factor (RF) of max. 20% were considered.

**Results:**

Perimetry follow-up of 101 eyes in 70 patients fulfilled the criterion of a max. 20% RF. Follow-up was performed on average 362.2 days after ONS. MD significantly decreased from PRE 14.0 dB (median) to POST 13.4 dB (*p* < 0.01). 64 eyes in 49 patients showed constant or reduced MD as compared to baseline (PRE 13.4 dB vs. POST 11.2 dB). In 37 eyes of 30 patients, MD increased from PRE 14.9 dB to POST 15.6 dB.

**Conclusions:**

Innovative treatments that preserve visual function through mechanisms other than lowering IOP are required for glaucoma with progressive vision loss. The present long-term data document progression halt in more than 63% of affected eyes after ONS and, thus, extend existing evidence from clinical trials.

## Background

Glaucoma is characterized by progressive vision loss culminating in blindness due to axonal degeneration and loss of retinal ganglion cells (RGC) (Schuster et al. 2020; Weinreb et al. 2014). Despite therapeutic reduction of intraocular pressure (IOP), patients may suffer from progressive vision loss (Anderson et al. 2001; De Moraes et al. 2017; Garway-Heath et al. 2015; Group CN-TGS 1998; Heijl et al. 2009; Heijl et al. 2002; Quigley 2012). Meta-analyses of glaucoma clinical trials assessing progression of visual field loss under effective IOP-lowering treatment suggested 0.54 dB/year as an estimate of the MD worsening rate in visual field testing for all glaucoma patients (De Moraes et al. 2017; Quigley 2012). Patients over 65 years of age as well as patients suffering from pseudoexfoliative glaucoma (PEX) were set apart by even faster rates of progression, with values amounting to 0.74 dB/year and 1.56 dB/year, respectively (De Moraes et al. 2017; Quigley 2012).

The IOP is considered to be the most important modifiable risk factor for glaucoma onset and progression (De Moraes et al. 2017; Schuster et al. 2020; Weinreb et al. 2014; Wojcik-Gryciuk et al. 2016). Correspondingly, the current standard approach in glaucoma therapy is IOP reduction, which does not totally halt progression, but significantly improves the visually functional lifespan of many patients (De Moraes et al. 2017; Heijl et al. 2009; Quigley 2012; Schuster et al. 2020). However, despite effective medications or surgical treatment leading to IOP-lowering, glaucoma exacerbation and progressive vision loss among patients is common (De Moraes et al. 2017; Garway-Heath et al. 2015; Group CN-TGS 1998; Heijl et al. 2002; Quigley 2012). Therefore, treatments that preserve visual function by protecting retinal structures as ganglion cells through mechanisms other than lowering IOP are required and would be a major breakthrough (Guymer et al. 2019; Khatib and Martin 2017).

Electrical stimulation is suggested to be a promising treatment option for diseases of the optic nerve and the retina (Fu et al. 2015; Rahmatnejad et al. 2016). Preclinical insights into neurorestorative and neuroprotective effects of electrical retinal stimulation triggered first translational clinical studies in patients (Gall et al. 2016; Ota et al. 2018; Rock et al. 2017). A randomized, sham-controlled clinical trial applied transcutaneous electrical stimulation via supraorbital and infraorbital electrodes to the eyes of 82 patients suffering from optic neuropathies (Gall et al. 2016). Electrical stimulation provoked phosphenes as a biomarker for excitation of optic nerve axons. Ten optic nerve stimulation (ONS) sessions were applied daily for 10 consecutive weekdays. The primary outcome measure for efficacy was high-resolution perimetry assessed 2 days and 2 months after the treatment cycle. The treated group had a significant improvement in visual field of 24% as compared to baseline. This improvement persisted for at least 2 months. In a further study, transcorneal electrical stimulation sessions were conducted once a week for six consecutive weeks in 14 patients with primary open angle glaucoma (POAG) (Rock et al. 2017). Patients were randomized into three groups with no stimulation (sham), and electrical stimulation intensities below (subthreshold) or above (suprathreshold) individual phosphene thresholds. No statistically significant differences in visual fields between groups were reported. Additionally, transcorneal electrical stimulation was applied in five eyes of four male subjects with POAG every 3 months throughout a period of approximately 4 years (Ota et al. 2018). Suprathreshold stimulation provoking phosphenes was administered for a duration of 30 min in each quarterly session. Baseline MD values before electrical stimulation were 17.3 dB on average. There was a significant linear relationship between changes in MD values and the number of electrical stimulation sessions indicating partially restored visual fields (*p* < 0.01). It was concluded that electrical stimulation treatment may improve glaucomatous visual field defects in POAG. All three studies applied active ONS with intensities above phosphene thresholds, however, number of patients or eyes in treated groups (45 vs. 4 vs. 5) and duration of evaluation periods (2 months vs. 6 weeks vs. 4 years) were rather heterogeneous. Furthermore, preclinical studies indicated the importance of frequent daily ONS sessions and the relatively long time course of restorative effects in models of optic neuropathies (Fu et al. 2018; Kurimoto et al. 2010; Morimoto et al. 2010; Okazaki et al. 2008; Tagami et al. 2009). However, two of three published clinical trials suggested improved vision by electrical stimulation treatment.

The aim of this retrospective study was to collect real-world data in a large number of patients with refractory glaucoma and to evaluate long-term clinical efficacy of electrical ONS. The results will provide the basis for the design and the power analysis of a future randomized controlled trial.

## Methods

The retrospective study was approved by the Charité’s Ethics Committee and was conducted according to the 1964 Declaration of Helsinki (as amended by the 64th WMA General Assembly, Fortaleza, Brazil, October 2013).

Patients from the ophthalmology outpatient clinics in Berlin, Hannover, Ahaus, Neu-Ulm, and Munich, all in Germany, were included in the data analysis, if they fulfilled the following inclusion criteria:

1. Diagnosis of glaucoma with progressive vision loss despite appropriate IOP-lowering therapy.
2. Assessment of visual receptive field by static threshold perimetry in the central 30° with a reliability factor (RF) of max. 20% before ONS treatment (PRE).
3. Full ONS treatment cycle with 10 daily sessions.
4. Assessment of visual receptive field by static threshold perimetry in the central 30° with an RF of max. 20% approximately 1 year after ONS therapy (POST).

Patients could only opt for ONS treatment, if they were under appropriate IOP-lowering medication as monitored by Goldmann applanation tonometry. Thus, ONS did not replace drug treatment but was applied additionally in patients with progressive vision loss despite normal IOP values. No guidelines exist to determine whether structural or functional assessment is best suited to monitor glaucoma progression based on the presenting clinical signs (Abu et al. 2021). Consequently, expert ophthalmologists in clinical practice appraised visual field progression consulting patients’ evaluation of visual function and possibly various diagnostic findings from assessments such as perimetry, optical coherence tomography, or Heidelberg retinal tomography. This appraisal of progression of visual field impairment was at the discretion of expert ophthalmologists involved in the present retrospective study. Collected clinical data included age, sex, type of glaucoma, IOP, time frame of ONS treatment, date and outcome parameters mean defect (MD) and reliability factor (RF) of static threshold perimetries. MD was defined as primary outcome parameter.

### Perimetry

Patient’s visual receptive fields were assessed by static threshold perimetry in the central 30° applying the same perimeter device before and after ONS in the same patient (OCTOPUS, Haag-Streit Deutschland GmbH, Wedel, Germany). Whereas the MD was defined as primary outcome parameter, the RF served as the quality criterion of the perimetry and decided on the inclusion of patient’s data in the analysis. The MD was the average of all local defects as given in the comparison plot. All local defects in the respective visual field were considered. The MD is age-corrected (Weijland 2004). About 90% of normal visual fields have an MD in the range of − 2 to + 2 dB. MD is the most important index related to global glaucoma damage (Ohnell et al. 2017; Ohnell et al. 2016). A trend in visual field change can be analyzed best by following MD changes (Gillespie et al. 2003). Thus, perimetries were performed at baseline before ONS treatment (PRE) and at least once approximately 1 year afterward (POST). The RF indicated the patient’s cooperation. This value was calculated from positive and negative catch trial questions – the sum of the false positive answers and false negative answers, divided by the total number of catch trial questions (Weijland 2004). The upper limit of RF value was defined as 20%. If the RF was above 20%, the patient’s data were not included.

### Optic Nerve Stimulation (ONS)

The ONS treatment was conducted using the Eyetronic® device (Neuromodtronic GmbH, Potsdam, Germany) that applied electrical stimulation via goggles with embedded supraorbital and infraorbital electrodes and recorded EEG signals via an electrode cap. All four electrodes, two on each side, in the stimulation goggles were controlled by four separate constant-current stimulators with the following stimulation parameters:

- Pulse shape: biphasic, symmetric rectangular
- Pulse amplitude: up to 1.2 mA
- Pulse duration 14 to 20 ms
- Repetition frequency in pulse trains: 5 to 34 Hz

In the beginning of each treatment session, the individual patient’s α frequency was measured in the resting EEG. Subsequently, electrical phosphene thresholds were assessed separately for each of the four stimulation electrodes. Suprathreshold stimuli were applied with increasing repetition frequency to determine the flicker fusion frequency, i.e. the frequency at which intermittent stimulation appeared to be completely steady to the patient. During the actual therapy the selected stimulation channels were supplied by the electrical current and a frequency range that had been calculated from the assessments of electrical phosphene thresholds, α frequency, and flicker fusion frequency. One therapy session ran in six to eight series, each of them consisting of several cycles with changing frequencies and different numbers of pulses. Series were separated by short breaks of 1 minute. The daily duration of the actual treatment stimulation did not exceed 40 min and varied slightly from one treatment day to another. Daily stimulation sessions took about 80 min including setup of the medical device, assessments of thresholds and frequencies, and actual therapeutic stimulation. Patients participated in 10 daily treatment sessions within 2 weeks.

### Statistics

Patient’s data were analyzed by descriptive statistics calculating arithmetic mean (Mean), median (Median), and standard deviation (SD). Differences of MD values at PRE and POST time points were statistically investigated by the Wilcoxon signed rank test. The change of MD was calculated by subtracting the PRE MD value from the POST MD value and abbreviated to ΔMD. If ΔMD was 0 or negative the corresponding eye was categorized as a responder. If ΔMD was greater than 0 the corresponding eye was categorized as a non-responder. The responder rate equaled the percentage ratio of the responders to all eyes. Potential relationships between any pair of variables were analyzed by calculating Pearson product moment correlation. All statistics were performed by using SigmaPlot and SigmaStat software (Systat Software, Inc., San Jose, CA, USA).

## Results

Clinical data from 101 eyes in 70 patients (31 female, 39 male) fulfilled the inclusion criteria. Patients were 68.5 ± 10.4 years old (Mean ± SD) ranging from 45 to 86 years. 41% of patients were younger than 68 years. IOP was 12.3 ± 2.7 mmHg (Mean ± SD). The ophthalmologists of all involved clinical centers diagnosed the following types of glaucoma (number of eyes): primary open-angle glaucoma (POAG, 65), normal tension glaucoma (NTG, 13), angle-closure glaucoma (ACG, 6), pseudoexfoliative glaucoma (PEX, 6), juvenile glaucoma (4), pigmentary glaucoma (4), glaucoma fere absolutum (2), and congenital glaucoma (1). The RF of all corresponding perimetries was maximal 20%. ONS treatment cycles of 10 days duration took place in the period from June 2014 to July 2019. The perimetry follow-up was performed 362.2 ± 45.4 days (Mean ± SD) after the ONS treatment corresponding to approximately 1 year.

**Fig. 1:**
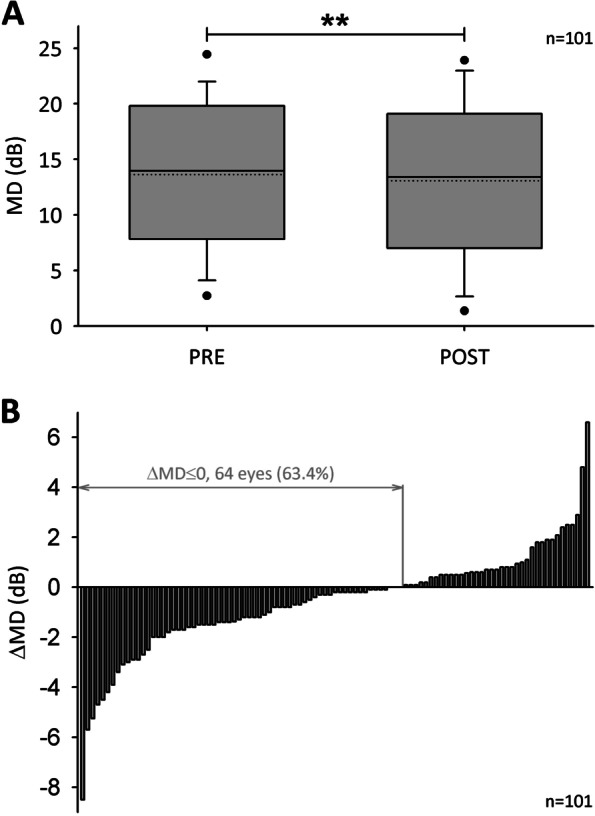
**A** Boxplots of PRE and POST mean defect (MD) in 101 eyes of 70 patients. Mean is given as dotted line. Black dots mark 5th and 95th percentiles. Asterisks indicate statistically significant reduction as analyzed by Wilcoxon signed rank test (*p* < 0.01). **B** Changes of MD in all 101 eyes as calculated by subtraction of PRE value from POST value (ΔMD). In 64 eyes of 49 patients (63.4%) ΔMD was 0 or negative

The baseline MD under PRE condition was 13.6 ± 6.9 dB with a Median of 14.0 dB. 89% of baseline MD values ranged from 2 to 22 dB. According to published classification of glaucoma severity, 20 (19.8%), 22 (21.8%), and 59 (58.4%) eyes were categorized as early (MD < 6 dB), moderate (6 dB ≤ MD ≤ 12 dB), and advanced glaucoma (MD > 12 dB), respectively (Proudfoot et al. 2021). One year after ONS treatment, the Median MD changed from 14.0 dB at PRE to 13.4 dB at POST (Fig. 1A). The Wilcoxon signed rank test suggested a statistically significant difference corresponding to an MD reduction within 1 year in all eyes (Z = -2.8, *p* < 0.01). The change of MD as calculated by subtraction of PRE value from POST value (ΔMD) was − 0.5 ± 2.1 dB ranging from − 8.5 dB to 6.6 dB (Fig. 1B). In 62 eyes of 46 patients older than 65 years, ΔMD was − 0.8 ± 2.2 dB.

**Fig. 2:**
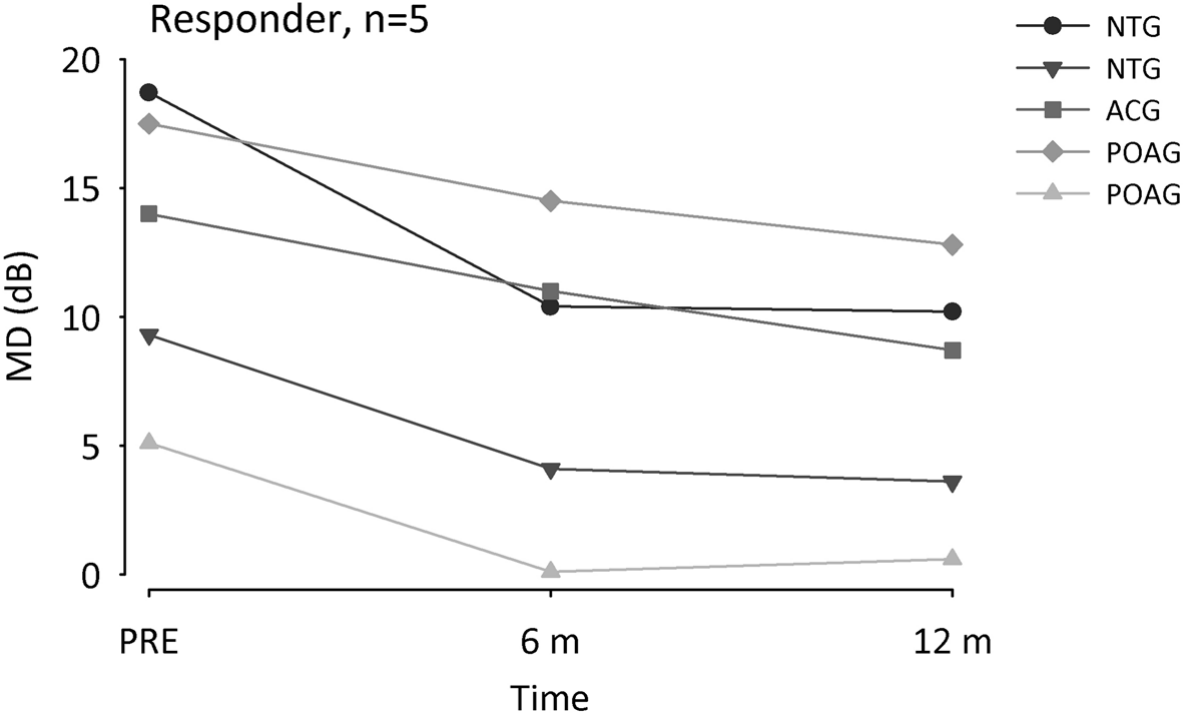
Line graphs of mean defect (MD) in five responders before optic nerve stimulation (PRE), and 6 and 12 months after treatment. Patients suffered from primary open-angle glaucoma (POAG), normal tension glaucoma (NTG), or angle-closure glaucoma (ACG)

**Fig. 3:**
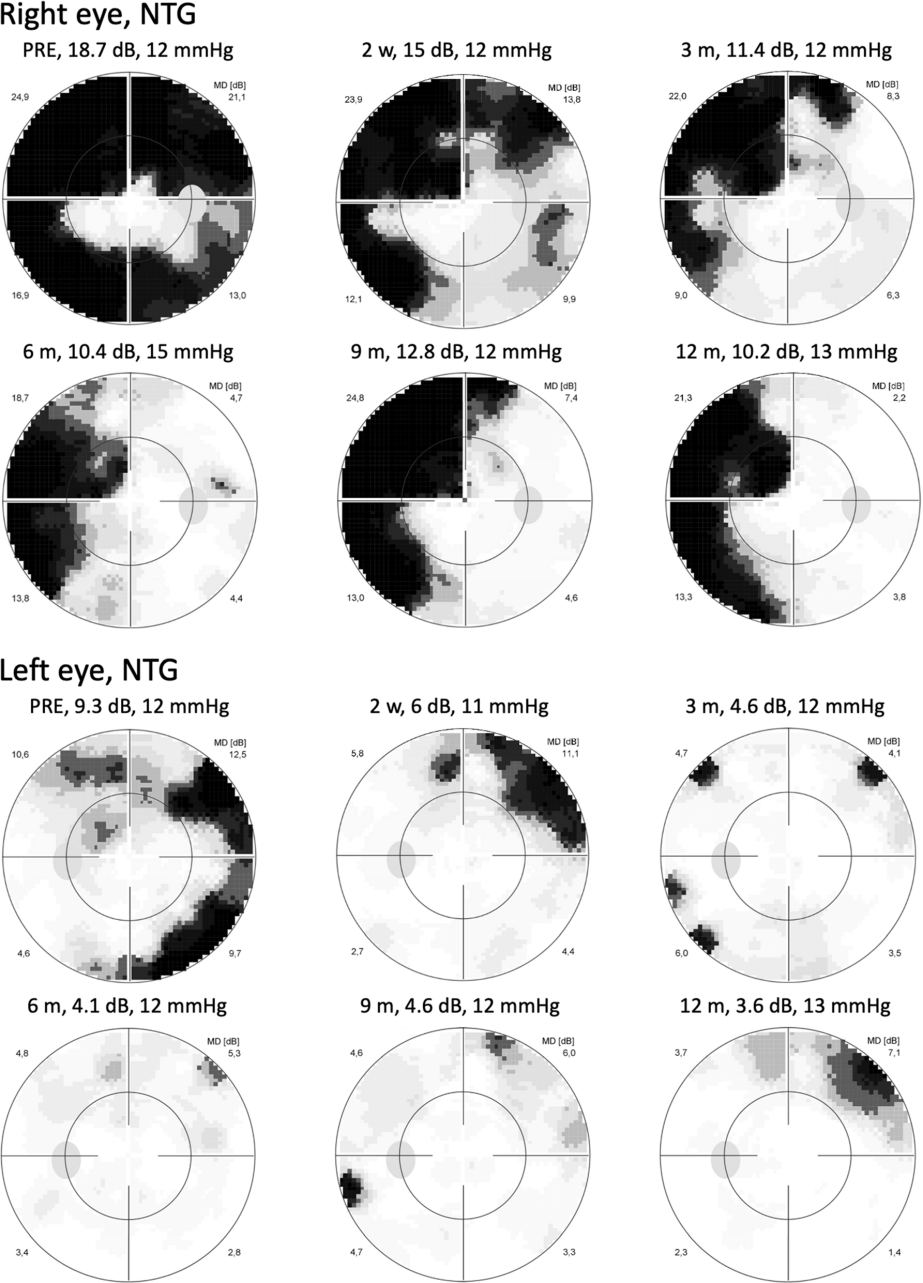
Visual fields of the right (top) and the left eye (bottom) with normal tension glaucoma (NTG) of a female patient, 70 years old. With each perimetry at time points PRE, 2 weeks, 3, 6, 9, and 12 months after ONS treatment mean defect (MD) and intraocular pressure are given

**Fig. 4:**
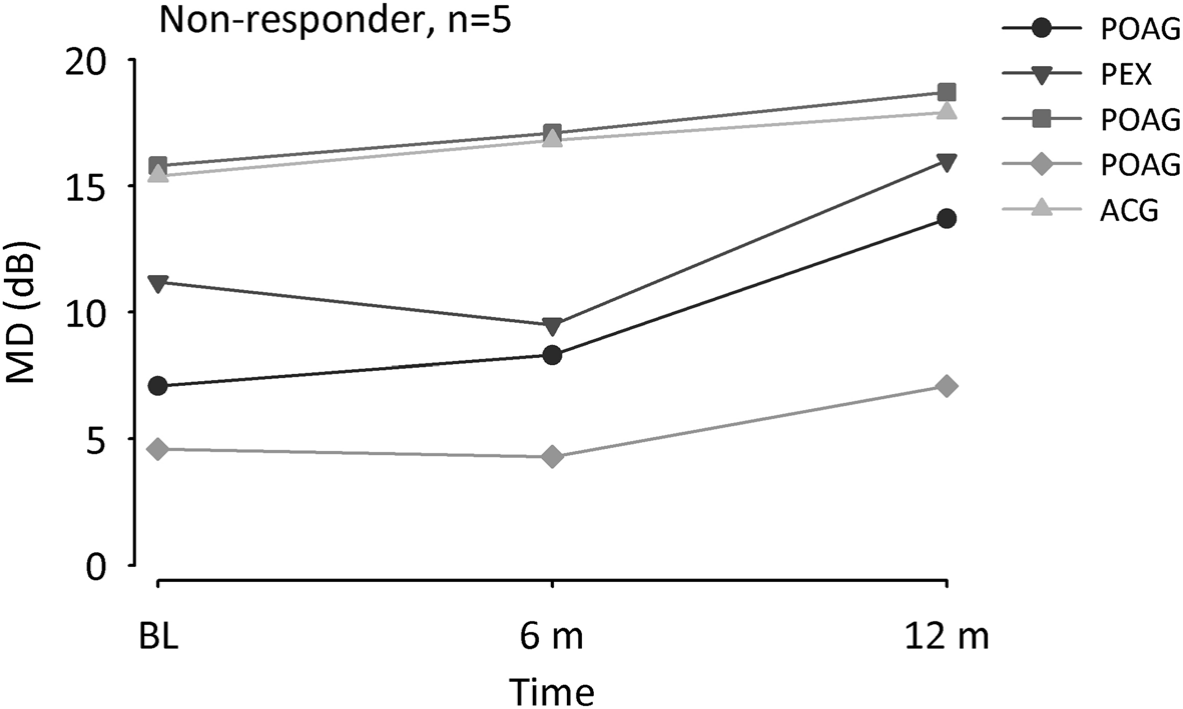
Line graphs of mean defect (MD) in five non-responders before optic nerve stimulation (PRE), and 6 and 12 months after treatment. Patients suffered from primary open-angle glaucoma (POAG), angle-closure glaucoma (ACG), or pseudoexfoliative glaucoma (PEX)

In 64 eyes of 49 patients (63.4%) ΔMD was 0 or negative indicating halt of vision loss progression or even a tendency to improvement within 1 year after ONS treatment (Fig. 1B). These responders had a baseline MD of 13.4 dB (Median) and 1 year after ONS an MD of 11.2 dB (Z = -6.8, *p* < 0.001). Non-responders with a ΔMD > 0 in 37 eyes of 30 patients showed an increase of MD from 14.9 dB (Median) at baseline to 15.6 dB under POST condition (Z = 5.3, *p* < 0.001). In five responders and non-responders each with extreme values of MD reduction or increase, respectively, additional perimetries at approximately 6 months after ONS treatment were available. The five top responders suffered from NTG, ACG, and POAG, started with baseline MD values between 5.1 and 18.7 dB and already showed a clear MD reduction 6 months after ONS (Figs. 2, 3). The five non-responders with the strongest MD increase 1 year after ONS were diagnosed with POAG, PEX, and ACG, and had PRE MD values from 4.6 to 15.8 dB. Whereas three patients already showed an MD increase 6 months after ONS, in two patients MD slightly reduced after 6 months before an increase occurred one year after ONS (Fig. 4).

**Fig. 5:**
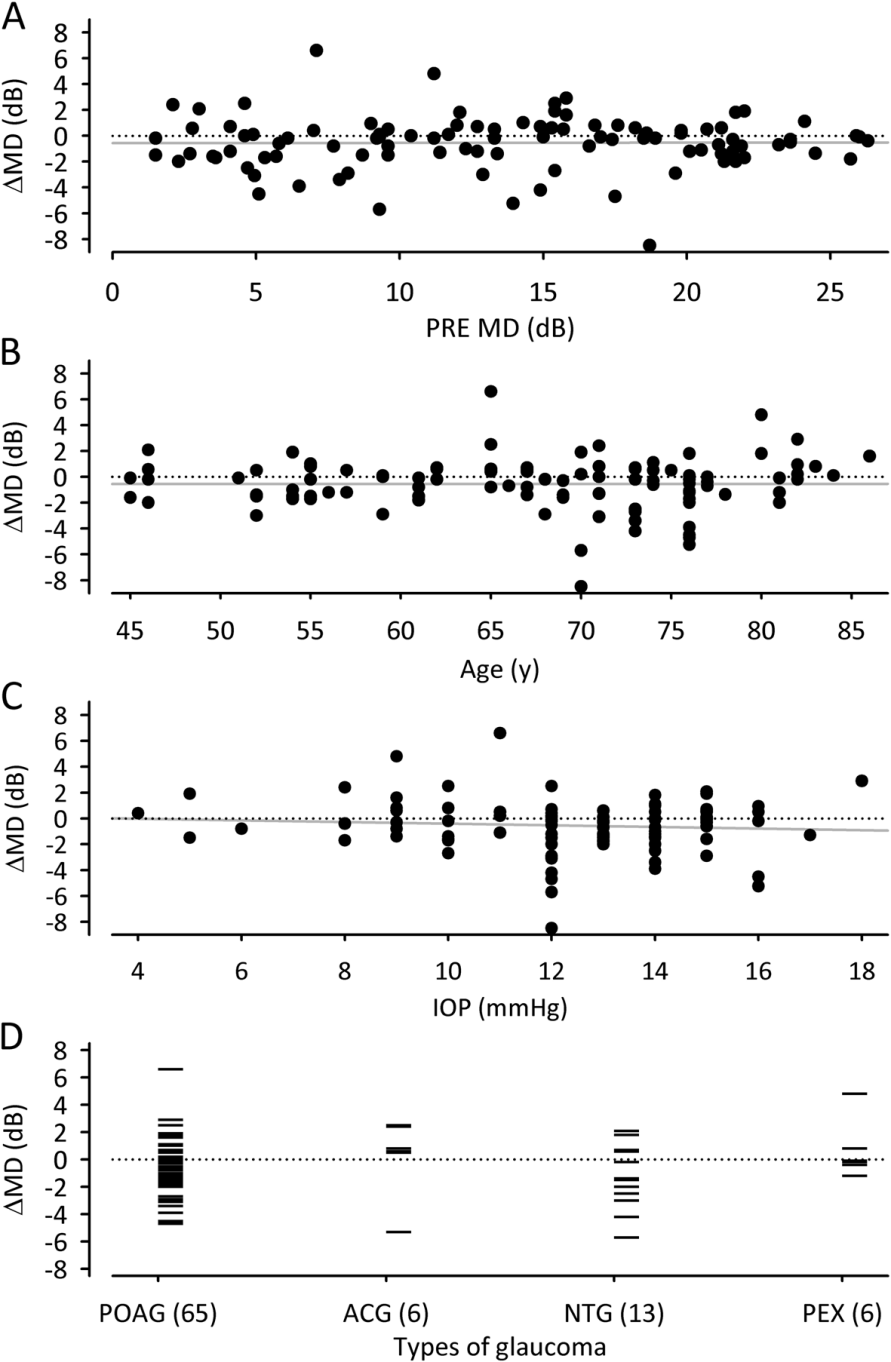
Scatter plots with linear regression analysis of PRE MD versus ΔMD (**A**), patients’ age versus ΔMD (**B**), and IOP versus ΔMD (**C**). Regression lines are given in gray. Vertical point plots of ΔMD in the four main types of glaucoma (**D**). Numbers of eyes in each group are presented in brackets. Primary open-angle glaucoma (POAG), angle-closure glaucoma (ACG). normal tension glaucoma (NTG), pseudoexfoliative glaucoma (PEX)

Changes of MD after ONS treatment may depend on various factors such as PRE MD, patients’ age, IOP, or types of glaucoma (Fig. 5). Potential relationships between baseline PRE MD, patients’ age, and IOP on one hand and ΔMD after 1 year on the other hand were analyzed by calculating Pearson product moment correlation. No statistical correlations were indicated. Qualitative comparison of ΔMD after 1 year in the four main types of glaucoma demonstrated similar responder rates of 69% in POAG and NTG eyes with average ΔMD of − 0.6 ± 1.8 dB and − 1.8 ± 3.0 dB, respectively. However, only six eyes each were diagnosed with ACG or PEX with average ΔMD of 0.3 ± 2.9 dB and 0.6 ± 2.1 dB, respectively.

## Discussion

The current study revealed a significant change of MD by − 0.5 dB/year on average in visual field testing in 101 eyes of 70 patients with progressive glaucoma, pointing to an overall improved vision in the majority of treated eyes. Median MD at baseline before ONS treatment was 14 dB, more than 58% of eyes had an advanced vision loss with an MD > 12 dB, more than 80% of eyes were classified as moderate or advanced glaucoma corresponding to an MD ≥ 6 dB.

All published clinical studies indicated progression of vision loss under best medical practice with IOP-lowering therapy even in early glaucoma (Garway-Heath et al. 2015; Group CN-TGS 1998; Heijl et al. 2002; Proudfoot et al. 2021). A cohort study of patients with open-angle glaucoma reported on 97 patients with an MD worsening rate of 0.32 dB/year (Proudfoot et al. 2021). The average MD of 4.3 dB under baseline condition was composed of 79, 12, and 9% of eyes with early, moderate, and advanced visual field loss, respectively. A retrospective chart review of 583 patients with manifest POAG or PEX glaucoma calculated visual field progression rates as slopes of MD over time (Heijl et al. 2013). Median MD at baseline was 10 dB, mean follow-up time was 7.8 years. Overall average MD progression rate was 0.8 dB/year, 5.6% of eyes progressed at rates worse than 2.5 dB/year. Glaucoma progression with MD worsening was observed in 89% of patients. These real-world data, covering a wide range of glaucoma severity with a Median MD of 10 dB, complemented progression rates from other clinical trials, which primarily recruited newly diagnosed patients with open-angle glaucoma under treatment and a baseline MD at 3.3 to 4 dB (Garway-Heath et al. 2015; Heijl et al. 2013; Heijl et al. 2002). Even those studies with mainly early glaucoma patients reported on progressive vision loss in 20 to 45% of cases with MD worsening rates between 0.4 to 0.8 dB/year. The worsening rate of about 0.5 dB/year in NTG patients with an MD of 8 dB at baseline resembled values from other studies (Anderson et al. 2001; Group CN-TGS 1998). Real-world data from more than 580 patients with mainly moderate and advanced glaucomatous vision loss provided evidence for further progression in the vast majority of patients amounting to 89% (Heijl et al. 2013). In contrast, present perimetry data in mainly moderate and advanced glaucoma under IOP-lowering therapy emphasized halt of progression or even improved vision in more than 63% of 101 eyes.

Especially patients over 65 years of age and patients suffering from PEX glaucoma were suggested to have even faster rates of progression with 0.74 dB/year and 1.56 dB/year, respectively (De Moraes et al. 2017; Quigley 2012). Current data calculated a mean MD change of − 0.8 dB/year in 62 eyes of patients older than 65 years and 0.6 dB/year in 6 eyes with PEX glaucoma. In comparison to published worsening rates in the elderly, current MD changes correspond to an improvement instead of a worsening of visual fields and, therefore, seem to substantiate the potential trend reversal.

Assessment of visual fields was performed by static threshold perimetry in 24° or 30° of the central region in those clinical trials that focused not only on the primary outcome parameter IOP, but also on clinically relevant visual field loss (Anderson et al. 2001; Garway-Heath et al. 2015; Group CN-TGS 1998; Heijl et al. 2013; Heijl et al. 2002; Proudfoot et al. 2021). Perimetry is an established and approved psychophysical method with sound evidence for high sensitivity in detection of progressive vision loss (Ohnell et al. 2017; Ohnell et al. 2016). The Early Manifest Glaucoma Trial (EMGT) addressed the temporal relationship between detection of glaucomatous optic disc progression, as assessed by fundus photography, and visual field progression in 306 eyes (Ohnell et al. 2016). Progression was detected in the visual field first in 163 eyes, more than 4 times as often as progression in the optic disc. The assessment of 210, 83, and 13 eyes with early, moderate, and advanced visual field loss, respectively, showed that the progression was detected first in the visual field in 80, 79 and 100%, respectively (Ohnell et al. 2017). Thus, the initial progression was detected much more often in the visual field series than in the optic disc photographs at all stages of disease. Relationship between visual field testing and RGC count was addressed by various approaches observing a nonlinear relationship between the MD in standard automated perimetry and cell counts (Hood 2019; Medeiros et al. 2013; Medeiros et al. 2012; Quigley et al. 1989; Torres and Hatanaka 2019). The association between severity of visual field loss and self-reported health-related quality of life (HRQOL) was investigated in 5213 participants (McKean-Cowdin et al. 2007; McKean-Cowdin et al. 2008). MD scores were used to determine severity of visual field loss, HRQOL was assessed by the Medical Outcomes Study 12-item Short-Form Health Survey (SF-12) and the National Eye Institute Visual Function Questionnaire (NEI-VFQ-25). Worse NEI-VFQ-25 and SF-12 HRQOL scores were associated with visual field loss in a linear manner. Four to 5 dB differences in MD were associated with a 5-point difference in the NEI-VFQ-25. Thus, static threshold perimetry and its key parameter MD are approved tools to assess visual field loss with clear evidence of relationships with RGC count and quality of life (Jammal et al. 2019).

RGC apoptosis is the final common pathway causing vision loss in glaucoma. Various and finally lethal injuries to RGC and their axons trigger stressful changes of the cellular and molecular environment that exceed their survival capacity (Porciatti and Ventura 2012). A hypothesis suggests that the early stages of optic neuropathies are characterized by failure of physiological mechanisms such as axonal transport to sustain normal cell function under prolonged exposure to hazards (Erb 2012). Autoregulatory failure gears adaptive mechanisms to prolong cell survival. Surviving RGC have altered function, which may be reversible under less stressful conditions. The duration of the stage of cell dysfunction preceding death may be relatively long in glaucoma. The therapeutic time window between the beginning of altered cell function under stress until degeneration of structure, i.e. apoptosis, provides the opportunity to apply innovative therapeutic approaches to prevent RGC death and restore cell function. Progressive glaucoma probably is characterized by simultaneous occurrence of various stages of cell function and structure in the retina. The main target for innovative treatments in glaucoma are those RGC that are set under stressful conditions with loss or altered function and maintained structure.

Studies in animal models of optic neuropathies suggest the hypothesis that electrical stimulation of RGC is able to trigger various mechanisms of action that support the transition from dysfunctional cells with preserved structure to healthy functional cells as a prerequisite for physiological visual processing (Fu et al. 2018; Fu et al. 2015; Hanif et al. 2016; Rahmatnejad et al. 2016; Yin et al. 2016). Preclinical studies investigated potential regenerative and protective effects of electrical stimulation in animal models of optic neuropathies such as glaucoma. After a standardized optic nerve crush, electrical stimulation of the eye promoted both axonal regeneration and survival of RGC in a dose-dependent manner (Tagami et al. 2009). Functional impairment of the optic nerve was assessed by visual evoked cortical potentials in rats after optic nerve crush (Miyake et al. 2007). Whereas no spontaneous recovery of evoked potentials occurred in the control group within 1 week after the crush injury, electrical stimulation of the eye for 6 h immediately after the crush caused partial restoration of visual evoked potentials and protected retinal axons from imminent degeneration. Transection of the optic nerve in rats reduced the number of RGC down to approximately 50% within 1 week (Morimoto et al. 2005). One hour of electrical stimulation of the eye right after optic nerve transection was able to significantly protect RGC from apoptosis. Acute ocular hypertensive injury with 80 mmHg for 1 hour was applied to gerbil eyes (Fu et al. 2018). In the treatment group, electrical stimulation was applied to the eye immediately after the injury and then twice a week for a total of 1 month. Electrically stimulated eyes had a significantly higher survival of RGC after 1 month when compared to the control group. Experimental studies in various disease models addressed mechanisms of action and indicated modulation of neurotrophic factors (IGF-1, BDNF, CNTF, FGF-2, TNF-α) and immunomodulators (IL-10, IL-6, COX-2, NF-κB) by electrical stimulation of the eye in optic neuropathies (Fu et al. 2018; Fu et al. 2015; Hanif et al. 2016; Yin et al. 2016).

The main limitations of the present study are the retrospective design of real-world data assessment and the sole application of the visual receptive fields. The analysis of retrospective data depended on common clinical practice. Thus, data assessment was determined by clinical requirements and did not follow a specified clinical investigation plan. Due to these real-world data characteristics, in all 101 eyes of 70 patients visual field testing with identical parameters was available for at least two points in time, at baseline just before ONS and after approximately 1 year. Additional perimetry assessments with identical parameters in some patients were scattered and sporadic and did not qualify for a statistical analysis in all patients. Visual field testing and many other psychophysical assessments share the potential variability of data. Consequently, this feature of perimetry data is considered by the application of the Wilcoxon signed-rank test. This statistical test is a nonparametric test that can be used to determine whether two dependent samples were selected from populations having the same distribution. Random variability would not explain the statistically significant reduction of MD over time. Learning effects have to be taken seriously. The vast majority of included patients suffered from glaucoma for many years or even decades and experienced the procedure of perimetry many times. Knowing the epidemiology of glaucoma, the duration of illness is substantiated by patients’ age of 68.5 ± 10.4 years. No newly diagnosed patient participated in the study. The average RF was 7.6% before stimulation and 6.3% after 1 year. Statistical testing did not indicate any significant change over time.

IOP-lowering by drugs and medical devices still dominate current glaucoma therapy (Schuster et al. 2020; Weinreb et al. 2014). Correspondingly, regulatory approval of new therapies in glaucoma has historically used IOP as the outcome variable (De Moraes et al. 2017; Quigley 2012). The average IOP of 12.3 mmHg in the present study was lower than those values in treatment groups of published randomized controlled trials ranging from 14.6 to 19.3 mmHg (Garway-Heath et al. 2015; Heijl et al. 2002; Kass et al. 2002). Due to progressive visual field loss in a considerable number of patients, treatments that preserve visual function through mechanisms other than lowering IOP are required. Such treatments need other primary outcome parameters such as visual field testing by perimetry and appropriate study designs (De Moraes et al. 2017; Proudfoot et al. 2021; Quigley 2012). A 30% decrease in visual field progression rate over 12 to 18 months in patients with glaucoma was suggested to be clinically meaningful, and therefore would be valuable for treating glaucoma patients who are progressing despite IOP-lowering therapy (De Moraes et al. 2017). Thus, standard clinical trials with two parallel groups, two-sided statistics, and potential decrease of visual field progression rate by 30%, would require 300 to 500 patients per group to be included with a follow-up between 12 and 18 months (Proudfoot et al. 2021; Quigley 2012). A trend reversal of worsening rates with significant improvement of visual fields by electrical ONS would significantly reduce the number of patients in a pivotal clinical trial with an evaluation period of 12 months until the primary endpoint.

Neuromodulation by electrical stimulation of nervous tissue has been introduced to clinical medicine many decades ago and became indispensable in various indication areas. Especially patients suffering from drug resistant diseases such as epilepsies, Parkinson’s disease, depression, and pain benefit from a broad spectrum of neurostimulation techniques such as vagus nerve stimulation, deep brain stimulation, spinal cord stimulation, and cortical stimulation (Antal et al. 2017; Ellrich 2019, 2020; Garcia et al. 2021; Gunduz et al. 2015). In 1755, Le Roy provoked phosphenes in a blind patient by sending electric current pulses through a wire wound around the head (Paulus 2010). Since then, various techniques have been established in order to apply electrical stimulation to the retina and the optic nerve via transcutaneous, transpalpebral, transcorneal, or transscleral applications (Colombo et al. 2021; Gall et al. 2016; Inomata et al. 2008; Rock et al. 2017). Safety data indicated non-significant risks associated with electrical stimulation of the eye (Antal et al. 2017; Fu et al. 2015; Rahmatnejad et al. 2016). Transcutaneous ONS in about 760 patients with optic neuropathies was accompanied by temporary adverse events such as skin sensations and irritation, headache, drowsiness, and sleep disturbances (Antal et al. 2017). Neither device-related serious adverse events nor incidents were reported. In humans, electrical stimulation of the eye has not been associated with major complications in over 33,200 sessions and 1000 patients (Rahmatnejad et al. 2016). It was concluded that electrical stimulation as a noninvasive, repeatable treatment modality with few reported side effects, could potentially be used to treat a variety of ophthalmological diseases (Fu et al. 2015; Rahmatnejad et al. 2016).

The present results will provide the basis for the design and the power analysis of a future randomized controlled trial. Such a trial will mainly address glaucoma patients with progressive vision loss as documented by repetitive and standardized threshold perimetry during an appropriate baseline period before application of ONS. Approximately 300 patients will be randomized in two parallel groups: Both groups will be treated by best medical practice, only 50% of patients will receive neurostimulation. During the evaluation period of at least 12 months threshold perimetry will be performed every 3 months. A subsequent open label extension will provide long-term safety and efficacy data.

## Conclusions

Innovative treatments that preserve visual function through mechanisms other than lowering IOP are required for glaucoma with progressive vision loss. The present long-term data document progression halt in more than 63% of affected eyes after ONS and, thus, extend existing evidence from clinical trials.

On a more general note, a variety of ophthalmological diseases may qualify for approaches of bioelectronic medicine. Case reports and small cohort studies suggest improvements of visual fields in patients suffering from retinitis pigmentosa and optic neuropathies of various origin, e.g. ischemic, hereditary, traumatic, inflammatory. Even vision loss of central origin due to stroke, traumatic brain injury, and multiple sclerosis may benefit from neurostimulation.

## Data Availability

Upon request.

## Abbreviations

ACG: Angle-closure glaucoma
CNTGS: Collaborative Normal Tension Glaucoma Study
EMGT: Early Manifest Glaucoma Trial
IOP: Intraocular pressure
MD: Mean defect
NEI-VFQ-25: National Eye Institute Visual Function Questionnaire
NTG: Normal tension glaucoma
ONS: Optic nerve stimulation
PEX: Pseudoexfoliative glaucoma
POAG: Primary open angle glaucoma
RF: Reliability factor
RGC: Retinal ganglion cells
SF-12: 12 item Short-Form Health Survey
UKGTS: United Kingdom Glaucoma Treatment Study
